# Experimental and Analytical Investigation of Self-Centering Precast Composite Walls with Sloped Plane Friction Dampers

**DOI:** 10.3390/ma17061319

**Published:** 2024-03-13

**Authors:** Wei Huang, Zhenhui Fan, Kang Liu, Gaoxing Hu, Xinwei Miao, Yujiao Sun, Gang Liu

**Affiliations:** 1 School of Civil Engineering, Xi’an University of Architecture & Technology, Xi’an 710055, Chinayujiaosun2000@163.com (Y.S.); ganglxauat@163.com (G.L.); 2 SCEGC NO.5 Construction Group Co., Ltd., Xi’an 710032, China; 3 School of Civil Engineering, Anhui Polytechnic University, Wuhu 241000, China

**Keywords:** self-centering, precast wall, sloped plane friction damper, steel wall toes, energy dissipation

## Abstract

To reduce the damage to reinforced concrete shear walls in earthquakes and repair costs, a self-centering wall with sloped plane friction dampers (SPFDs) is proposed. In addition to the SPFDs, the proposed wall includes a precast composite wall, steel wall toes, and post-tensioned (PT) tendons. The steel wall toes embedded in the base of the precast wall were used to improve its strength, and the SPFDs installed in the steel toes were used to increase its energy dissipation capacity. To investigate the effect of the initial PT force and prestressing clamping force of the friction bolt on the seismic performance of the wall, quasistatic cyclic loading tests were carried out on three precast wall specimens. The damage to the self-centering walls was slight, the residual drift was small, and the energy dissipation met the specification requirements. The wall with the greater initial PT force showed higher self-centering and bearing capacity, and the wall with higher prestressing clamping forces showed greater energy dissipation capacity. Additionally, a calculation method for the bearing capacity of the precast wall, which was verified by comparison with the test results, is suggested.

## 1. Introduction

Reinforced concrete (RC) walls are widely used as the primary lateral force-resisting components of middle- and high-rise buildings because of their strong stiffness and high strength. Compared with cast-in-place walls, precast walls have been widely studied and applied due to their energy savings, quick installation, and environmental protection [1,2,3]. The connection between the foundation or wall and the precast wall is usually a strong connection (e.g., a grout sleeve) [4,5] so that there are similar lateral bearing capacities and stiffnesses between the cast-in-place wall and the precast wall. Therefore, the damage to precast walls is similar to that to cast-in-place walls, which frequently involves the yielding of reinforcing bars and concrete crushing after a severe earthquake [5]. To avoid serious damage to the wall, Kurama et al. [6] developed a self-centering wall in which the wall and foundation were connected only by post-tensioned (PT) tendons. The test specimens showed slight damage and less energy dissipation.

To improve the energy dissipation capacity, a hybrid self-centering wall with energy dissipation devices was proposed. A small amount of ductile steel rebar placed between the wall and the foundation was developed to provide energy dissipation [7,8,9,10,11]. In recent years, the replacement of energy dissipation devices after yielding or damage has been widely studied. Marriott et al. [12,13] experimentally proposed a new self-centering wall with externally viscous fluid dampers, which can be replaced after an earthquake. Li et al. [14] proposed novel replaceable external buckling-restrained plates between the wall and foundation. Bedriñana et al. [15,16] developed new replaceable tension–compression yielding steel rod dampers outside of the wall. A new U-shaped steel plate placed at the wall base or at the vertical joint of two cross-laminated timber (CLT) walls was proposed to provide energy dissipation [17,18,19]. A novel steel oval-shaped damper [20,21,22,23,24] was developed to connect the wall to the rocking columns on both sides by welding, which dissipated energy by bending. Naserpour and Fathi [25] proposed a new O-shaped steel damper at the wall toes. East et al. [26] proposed novel externally mounted cantilevered steel flexural yielding arms in a steel rocking base at the bottom of the wall. Cui et al. [27] developed a novel alternative shear damper in the vertical joints of the coupled wall. Moghaddam and Shooshtari [28] proposed a multiple-slit metal damper between the self-centering wall and column. Wu et al. [29] proposed a novel metal plate at the wall toes.

The friction damper is another energy dissipation tool that can be easily replaced. Cui et al. [30] developed a novel self-centering hybrid shear wall system connecting the frame and wall with friction dampers. Liu et al. [31] developed a friction damper placed between the wall and foundation. Du et al. [32] developed a novel friction damper placed at the wall toes, which dissipates energy through friction during small drifts and increases energy dissipation through plastic deformation of the friction device during large drifts. Hashemi et al. [33] developed a novel resilient slip friction (RSF) joint composed of a saw-tooth configuration and disc springs to provide variable friction and self-centering capacity in a cross-laminated timber (CLT) wall. Tatar and Dowden [34] proposed a new variable friction damper composed of two angled steel wedges and disc springs in a CLT wall. Lepine Lacroix and Tang [35] proposed a novel self-centering friction damper placed in the vertical joints of two CLT walls. Darani et al. [36] developed a new self-centering friction damper placed at the wall toes or in the vertical joints of two walls. However, the self-centering friction damper would need to occupy a large amount of building space to provide sufficient self-centering and energy dissipation capacity for the RC wall.

To prevent the concrete at the wall toes from collapsing, researchers have proposed steel jackets [16] or rubber mats [24,25] at the wall toes. However, this results in energy damper devices being placed outside the walls and occupying more building space.

In this paper, a self-centering precast composite wall with sloped plate friction dampers (SPFDs) is proposed. The SPFDs provided sufficient energy dissipation without occupying too much building space, and the friction increased with the increase in wall drift. The steel wall toes improved the compressive strength of the wall and provided installation space for SPFDs. The composite wall exhibited a high strength and insulation effect. Three self-centering precast walls were tested under low-cyclic loading to examine their seismic behavior. Finally, a theoretical analysis of self-centering walls was carried out.

## 2. The Details of the Self-Centering Wall and SPFDs

**Figure 1:**
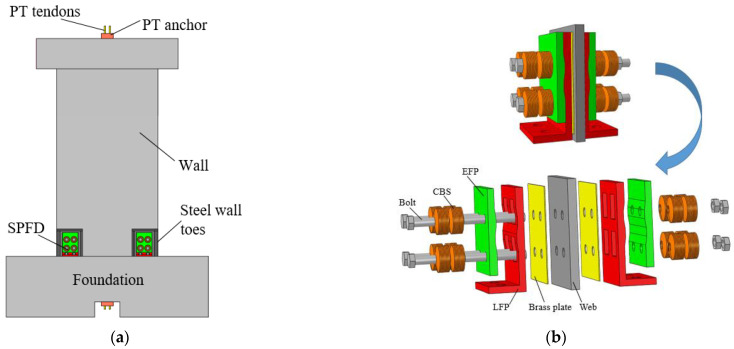
Self-centering precast composite wall with sloped plate friction dampers (SPFDs). (**a**) Configuration of the wall. (**b**) Details of sloped plate friction damper (SPFD).

The proposed self-centering wall is composed of a precast composite wall panel, PT tendons, steel wall toes, and SPFDs, as shown in Figure 1a. The composite wall exhibited a high bearing capacity, ductility, and deformation capacity. The PT tendons in the middle of the wall provided self-centering and bearing capacity. The steel wall toe could increase the strength of the wall base to avoid concrete crushing. The SPFDs, placed in the space between the flange and web, provided sufficient and flexible energy dissipation for the walls.

Figure 1b shows the details of the sloped plate friction dampers (SPFDs). As the wall toes rise, the external friction plate (EFP) slides along the slope of the L-type internal friction plate (LFP). The preload force given to the bolt of the combination of Belleville springs (CBS) assembly changes with the expansion of the EFPs, resulting in a change in friction force. The rectangular hole on the LFP prevented damage to the SPFDs caused by wall rocking. Brass panels provided stable energy dissipation for the walls.

## 3. Experimental Program

### 3.1. Specimen Description

Three self-centering precast walls with SPFDs (Construction by Matrix Construction Company in Xi’an, China) were subjected to testing under lateral cyclic loading, as shown in Table 1. Three specimens with identical dimensions on a 3/4 scale were designed and tested to obtain reliable experimental results, considering the limitation of laboratory space and budget constraints. The longitudinal reinforcing bars at the base of the wall were severed, and the wall connected to the foundation only through PT tendons and SPFDs. The thickness of the steel wall toes and walls was 150 mm, and the wall width was 1000 mm. The distance between the loading point and the wall base was 2000 mm, and the aspect ratio was 2 [37], which met the minimum requirement of 0.5 in ACI ITG-5.1 [37] to avoid shear-dominant damage.

**Figure 2:**
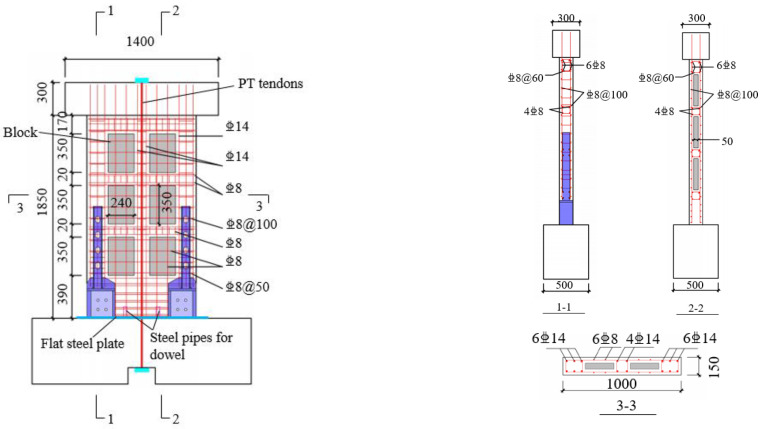
Reinforcement details of the test specimens (mm).

The specimen size and reinforcing bar details are identical, as shown in Figure 2. Each wall was composed of vertical and horizontal reinforcements. Six hexahedral spaces were inside the reinforcement cage for embedding the autoclaved aerated concrete blocks. These blocks played an important role in reducing self-weight and improving thermal insulation performance. To further improve the strength of the concrete at the base of the wall, each side of the rib column had 6 mm diameter longitudinal bars and transverse hoops spaced at 50 mm. Positioned in the middle of the wall were four PT tendons through a PVC pipe, each with a diameter of 15.2 mm. The unbonded PT tendons were used to provide the restoring force. To enhance the durability of PT tendons in engineering applications, the surface of these tendons should be coated with a specialized anti-corrosion lubricating grease, and the wrapping material should ensure continuous coverage along the entire length of the unbonded PT tendons [38]. The damage to PT tendons can be detected using methods such as ultrasonic testing, electrochemical testing, and electromagnetic testing. However, the existing technology for replacing unbonded PT tendons is not yet fully developed, and further research in this area could draw insights from advancements in bridge engineering [39].

**Figure 3:**
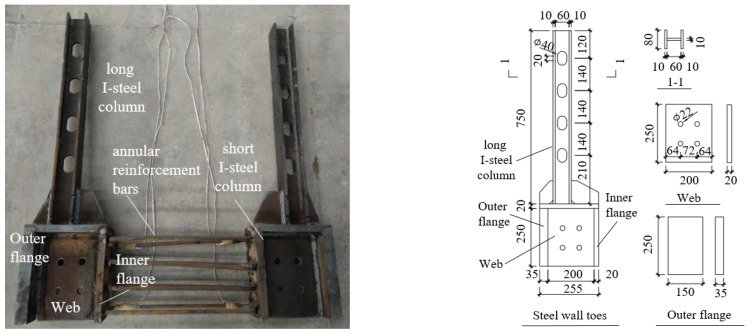
Details of the steel wall toes (mm).

As shown in Figure 3, the steel wall toes were composed of a long I-steel column at the upper part and a short I-steel column at the lower part. A long I-shaped steel column was embedded in the concrete to enhance the compressive and tensile strength of the wall. The outer flange width of the short I-steel was 150 mm, consistent with the wall thickness. The outer flange had a 35 mm thickness to prevent excessive buckling deformation during compression of the wall toes. The flange height was 250 mm to fit the sloped friction plate. The space of the steel wall toes was convenient for placing SPFDs while occupying little building space.

**Figure 4:**
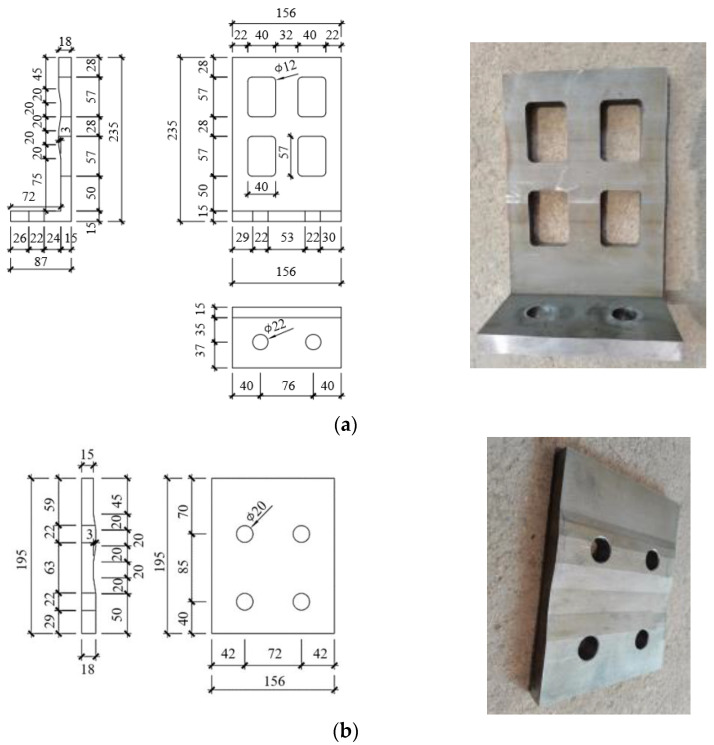
Details of the SPFD (mm). (**a**) LFP. (**b**) EFP.

The design of the SPFD is shown in Figure 4. The maximum thickness of the friction plates was 18 mm, and the minimum thickness was 15 mm. The convex part of the LFP and the concave part of the EFP were paired with each other. To avoid collisions between the edge of the LFP holes and the friction bolts during wall rocking, four rectangular holes were opened in the LFP. A 2 mm thick brass plate with four circular holes was placed between the steel toe web and the LFP to stabilize friction. According to tests by Hashemi et al. [40] and Guo et al. [41], the friction coefficients of steel–steel, *μ_L-W,_* and steel–brass, *μ_L-E_*, were 0.18 and 0.3, respectively.

Four of the disc springs were in parallel, and four were in series during testing. In principle, these combinations were used to meet the specified requirements of the energy dissipation capacity through prestressing clamping forces [37] and to minimize the space outside the building occupied by the SPFD. The initial torque, *T*, was exerted on the friction bolts of PW1, PW2, and PW3 at 44 N·mm, 44 N·mm, and 88 N·mm, respectively. This corresponds to prestressing clamping forces of 9.5 kN, 9.5 kN, and 19 kN.

**Figure 5:**
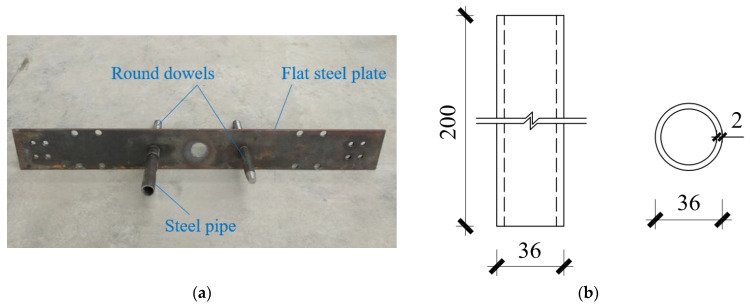
Details of steel pipes, round dowels, and flat steel plate (mm). (**a**) Physical figure. (**b**) Dimension of steel pipe.

Two steel pipes were embedded at the base of the wall and two Q345 round dowels were embedded at corresponding positions on the foundation, as shown in Figure 5. The inner and outer diameters of the steel pipes were 32 mm and 36 mm, respectively, and the diameter of the round dowels was 30 mm. When the wall was rocking, the round dowels prevented the horizontal movement of the steel pipes, thus preventing the horizontal movement of the wall bottom. Due to the high stiffness of the steel pipes, the deformation of the steel pipes was small; therefore, the surrounding concrete was not damaged under compression. A 10 mm thick flat steel plate was embedded on the upper surface of the foundation. When the wall was rocking, the area under compression at the bottom of the wall decreased rapidly, resulting in a rapid increase in stress on the foundation surface. Owing to the high stiffness of the flat steel plate, the deformation was relatively small, resulting in low compressive stress on the concrete. Therefore, the concrete on the upper part of the foundation avoided being crushed.

### 3.2. Construction Procedure

**Figure 6:**
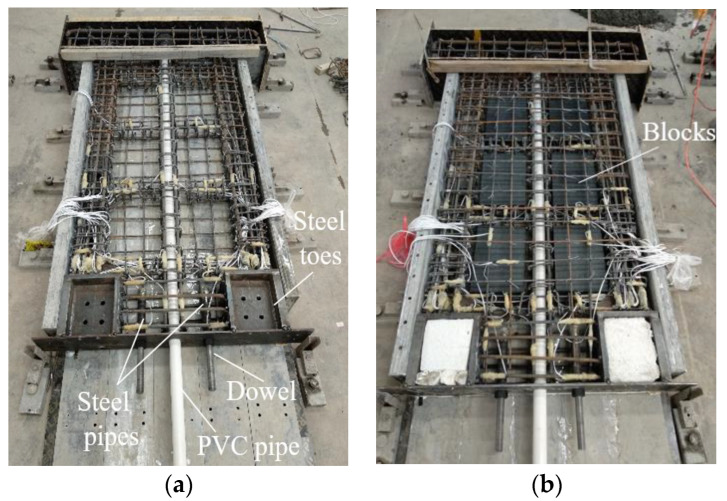

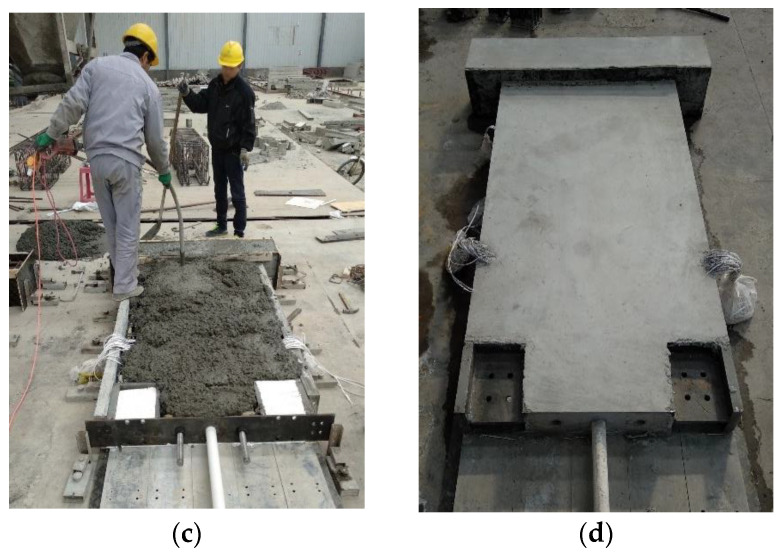
Wall fabrication. (**a**) Placement of reinforcement cage. (**b**) Placement of blocks. (**c**) Pouring concrete. (**d**) Cured wall.

Figure 6 shows the construction process of precast walls. The reinforcement cage was placed in the wall formwork, and the steel wall toes, steel pipes, round dowels, and PVC ducts were placed in corresponding positions. Following the casting of 50 mm concrete, autoclaved aerated concrete blocks, previously soaked for 12 h, were placed in the space left by the reinforcement cage. Then, distributed reinforcement mesh was placed on the upper side of the reinforcement cage. After recasting and adequate vibration, the walls were cured under standard conditions for 28 days.

### 3.3. Materials

The average compressive strength of concrete [42] for each specimen was 36 MPa. The properties of the reinforcing bars and steel plates were measured by tensile tests [43] and are given in Table 2. The diameter of the PT tendons was 15.2 mm. The Belleville spring material was 60Si2MnA and 50CrVA. The outer diameter and inner diameter of the Belleville springs are 60 mm and 20.5 mm, respectively, with a thickness of 3 mm and a maximum compression of 1.7 mm.

### 3.4. Test Setup and Measurement System

**Figure 7:**
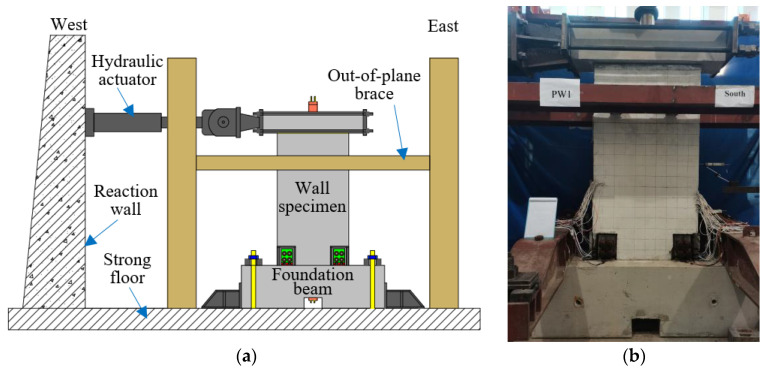
Test setup (mm). (**a**) Diagrammatic drawing. (**b**) Photograph of specimen.

To comprehensively study the seismic behavior of self-centering precast composite walls with SPFDs, including analysis of hysteresis curves, stiffness, energy dissipation, etc., quasistatic cyclic loading tests were conducted. Figure 7 shows the diagrammatic drawing and photograph of the test setup. In the designed structure [44], the self-centering wall only bore the vertical load from self-weight and PT force and did not bear the vertical load transmitted by the floor. The initial PT forces of precast wall PW1-3 were 300 kN, 407 kN, and 398 kN. The corresponding axial compression ratios are 0.102, 0.137, and 0.134, respectively. The axial compression ratio is the total axial force from self-weight plus the PT force divided by the wall’s effective cross-sectional area and the concrete compressive strength. The horizontal load applied to the loading beam was supplied through a horizontal hydraulic actuator.

**Figure 8:**
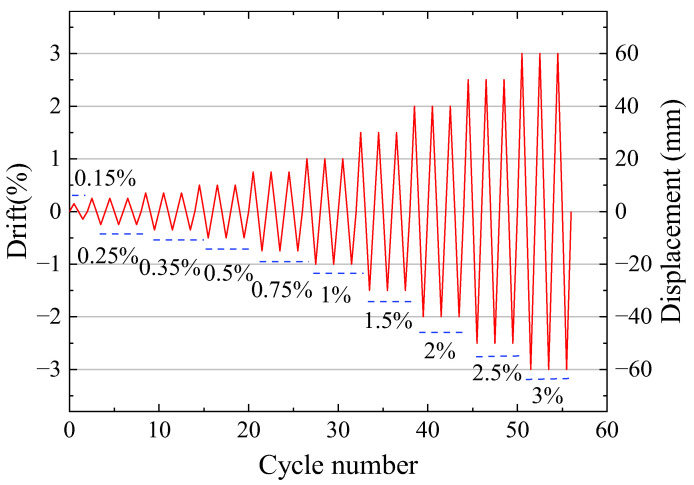
Loading history.

As depicted in Figure 8, except for 0.15% drift, the load applied to the walls included three fully reversed cycles at a level based on ACI ITG-5.1. Due to the limitation of the LFP holes, the test of the precast walls was terminated when the maximum drift of the wall reached 3%. However, this drift was greater than the minimum drift required in ACI ITG-5.1.

**Figure 9:**
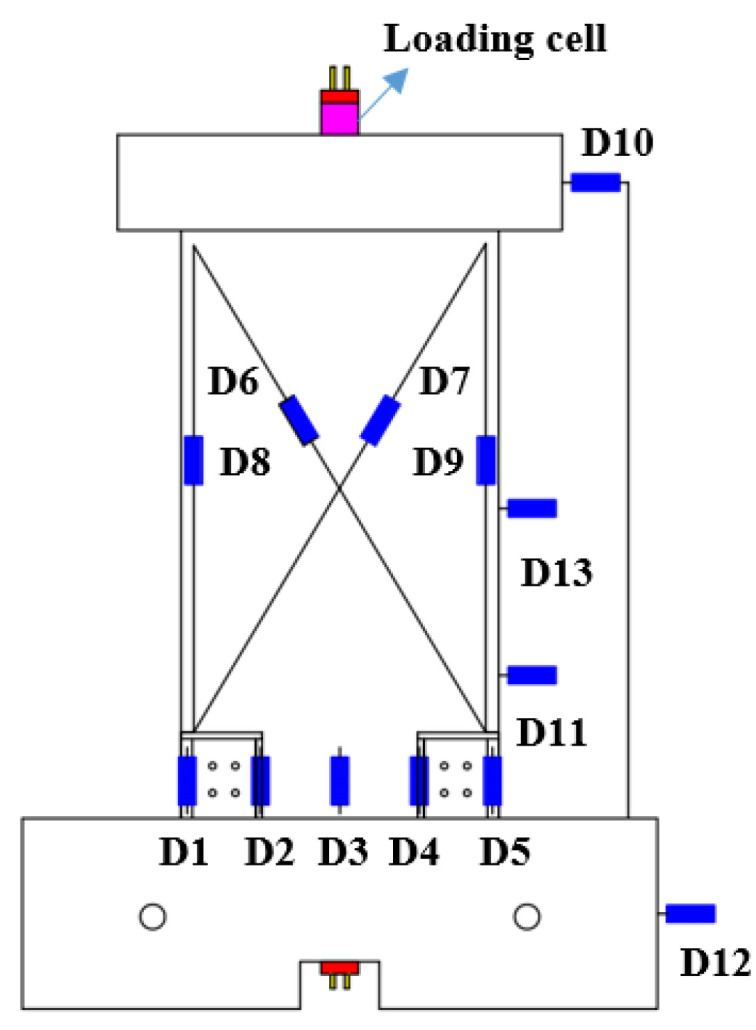
Arrangement of LVDTs.

As shown in Figure 9, horizontal linear voltage displacement transducers (LVDTs) (D10–D13), produced by Liyang Instrument Factory in Liyang City, China, were used to monitor the displacement of the specimen at different heights. Five LVDTs (D1–D5) were arranged at the bottom of the precast walls to measure the gap opening and uplift. Flexural deformations were measured by vertical LVDTs (D8–D9), while shear deformations were measured by intersecting LVDTs (D6–D7). The horizontal load and displacement were monitored by the horizontal actuator (MTS Systems Corporation, Minnesota, MN, USA). The force of the PT tendons was measured by a loading cell on the loading beam.

## 4. Results and Discussion

### 4.1. Failure Modes

**Figure 10:**
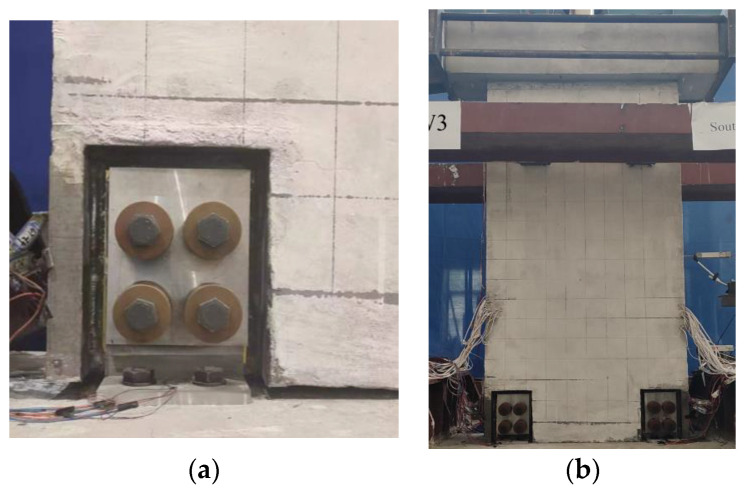
Test observations of specimen PW3. (**a**) Joint opening. (**b**) Final failure modes.

The test process of the three precast specimens was similar, and only PW3 was slightly damaged. Using specimen PW3 as an example, the horizontal joint at the bottom of the wall began to open at 0.25% drift. With increased drift, the gap gradually increased, and the relative slip of the SPFDs gradually increased, as shown in Figure 10a. To avoid collision between the bolt rod and the rectangular hole of the LFPs, the loading was stopped at 3% drift. As shown in Figure 10b, owing to the steel wall toes enhancing the strength of the wall toes, the wall was slightly damaged, and only a small amount of concrete peeled off at the bottom of the wall. The self-centering wall without a steel jacket displayed significant crushing at the wall toes [9,10,11,31].

### 4.2. Hysteretic Curves

**Figure 11:**
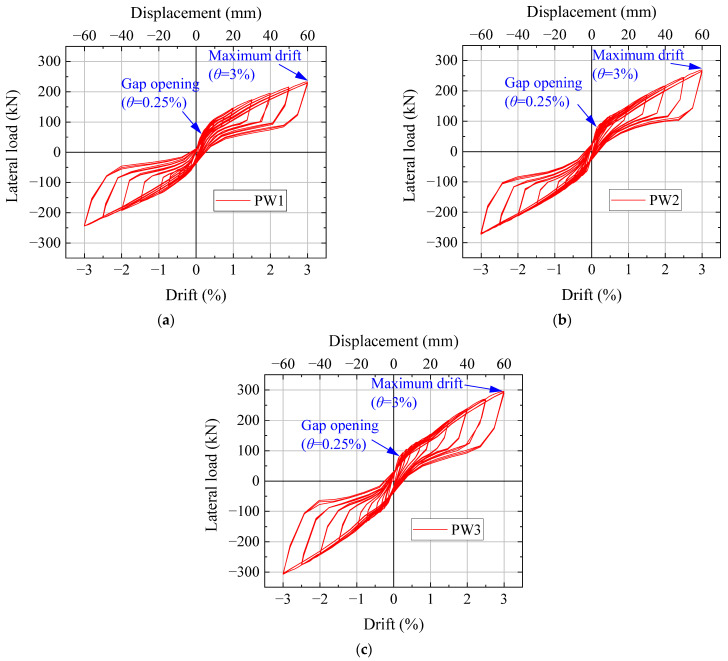
Hysteretic curves. (**a**) PW1. (**b**) PW2. (**c**) PW3.

The hysteretic curves of the three specimens are shown in Figure 11. The hysteretic curves of the precast specimens were flag-shaped. At 0.25% drift, the horizontal joint was opened, and then the stiffness decreased rapidly. The hysteresis loops of the specimens increased significantly with increasing drift because the compression of the CBS increased with increasing drift. The increase rate of energy dissipation in the wall with SPFDs was significantly higher than that in the wall without a damper and the wall with a flat plate friction damper [6,31].

PW2 and PW1 have the same initial prestressing clamping force of the CBS; therefore, the areas of the hysteresis loops were similar. Since the initial prestressing clamping force of the CBS of PW3 was greater than that of PW2, the initial compression amount of the CBS of PW3 was larger than that of PW2. The new compression amount of the CBS remained unchanged as the drift increased, leading to the total compression amount of PW3 being greater than that of PW2. Therefore, the hysteresis loops and lateral force of PW3 were larger than those of PW2.

### 4.3. Stiffness Degradation and Strength

**Figure 12:**
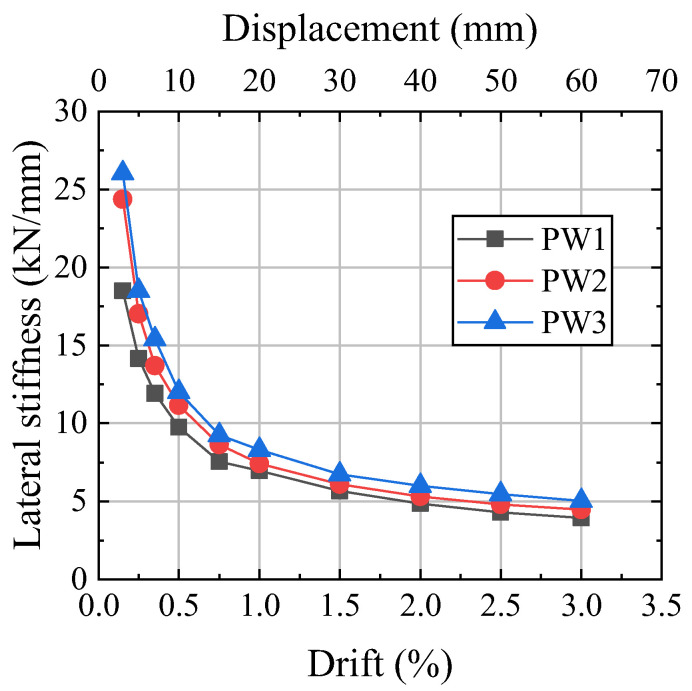
Stiffness degradation curves.

The stiffness degradation curves of the specimens are shown in Figure 12. The stiffness of the precast walls decreased obviously before 0.5% because the initial gap opening of the horizontal joints caused the contact area between the wall and the foundation to decrease rapidly. The stiffness decreased more slowly after 0.5% drift, which was related to slight wall damage, little PT force loss, and increased friction. As the drift increased, the stiffness was mainly determined by the increase in PT force, so the stiffnesses of the three specimens tended to be similar. Because the initial PT force of PW2 was 1.36 times that of PW1, the initial stiffness of PW2 was 1.32 times that of PW1. Due to the initial prestressing clamping force of PW3 being twice that of PW2, the initial stiffness of PW3 was 1.07 times that of PW2.

**Figure 13:**
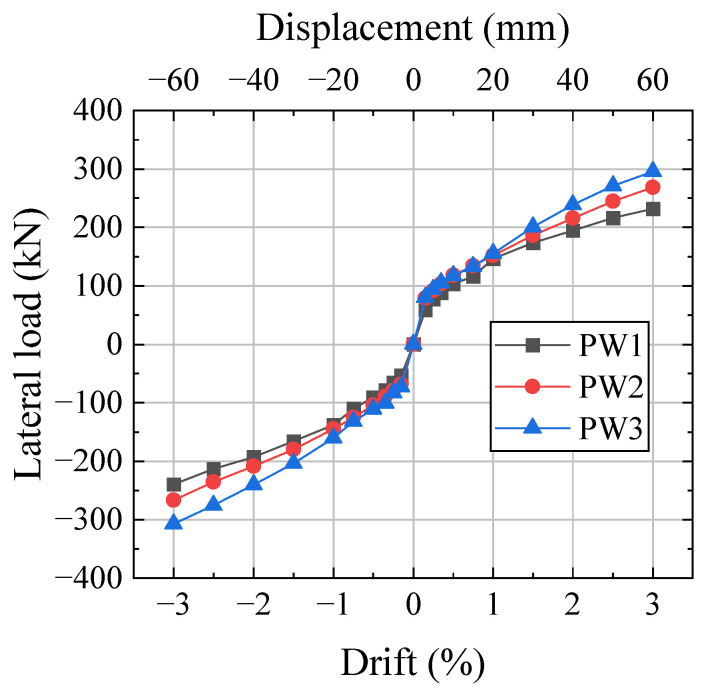
Skeleton curves.

The wall strength envelope of the cyclic response is shown in Figure 13. With increased drift, the strength of the specimens gradually increased, owing to the continuous increase in PT force and damper friction and slight damage to the wall. Due to the strengthening effect of steel wall toes, the strength of the wall did not decrease at large drift, while the wall without steel jackets experienced a decrease in strength at large drift due to the crushing at the wall toes [9,10,11]. Owing to the susceptibility of metals to damage during large deformations, certain walls with metal dampers experienced a decrease in strength due to the failure of these dampers at large drift [14,20,29]. Due to the initial PT force of PW2 being 1.36 times that of PW1, the PT tendons of PW2 provided greater lateral resistance than those of PW1 at the same drift; thus, the maximum load of PW2 was 1.16 times that of PW1. Due to the prestressing clamping force of PW3 being twice that of PW2, the SPFDs of PW3 provided a greater friction force; thus, the maximum load of PW3 was 1.10 times that of PW2.

### 4.4. Energy Dissipation Capacity

**Figure 14:**
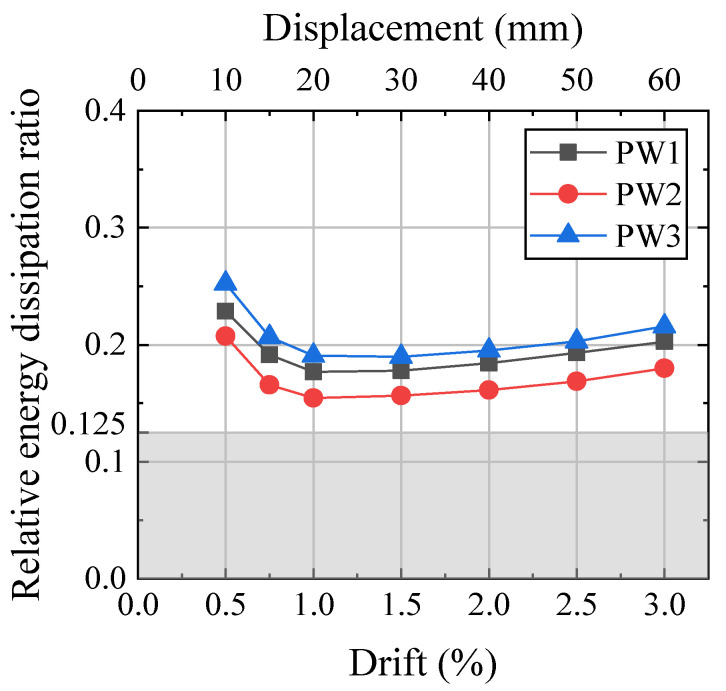
Relative energy dissipation ratio.

The relative energy dissipation ratio of the specimens is shown in Figure 14. Due to systematic friction, such as the friction caused by the nonparallelism between the LFP surface and the web of the steel toes and the friction between the PVC pipe and the PT tendons, the energy dissipation capacity in the early stage of loading was high. With increasing horizontal lateral force, the relative energy dissipation decreased first. Due to the rapid increase in the friction force of SPFD, the energy dissipation capacity of the wall gradually increased after 1.0% drift. The relative energy dissipation ratios of all precast walls met the requirements of 0.125 in ACI ITG-5.1. The relative energy dissipation ratio of the wall without a steel jacket decreased at large drift due to the crushing of concrete at the wall toes [9,10]. Due to the susceptibility of metals to damage during large deformations, the relative energy dissipation ratio of certain walls with metal dampers decreased at large drift [14,20].

Due to the initial PT force of PW2 being 1.36 times that of PW1, its lateral load was greater than that of PW1; therefore, the energy dissipation capacity of PW2 was 90% of that of PW1. Due to the prestressing clamping force of CBS in PW3 being twice that of PW1, the energy dissipation capacity of PW3 was 1.22 times that of PW2.

### 4.5. Residual Drift

**Figure 15:**
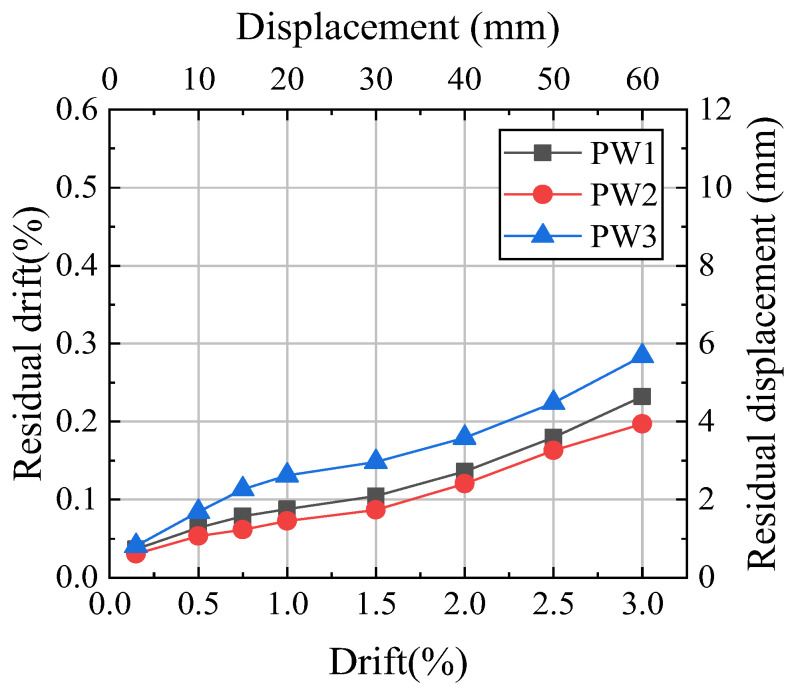
Residual drift.

The residual drift was one of the important parameters for evaluating the self-centering capacity of the wall. The residual drift of the precast walls is shown in Figure 15. Due to the susceptibility of metals to damage during large deformations, the residual drift of certain walls with metal dampers increased slowly or even decreased at large drift [14,20,29]. Due to the initial PT force of PW1 being 73% of PW2, the self-centering load of PW1 during unloading was smaller, resulting in a maximum residual drift of 1.17 times that of PW2. The prestressing clamping force of PW3 was twice that of PW2, and PW3 was subjected to greater resistance during unloading, resulting in a maximum residual drift of 1.44 times that of PW2. The initial prestressing clamping force and initial PT force greatly influenced the residual drift of the self-centering wall.

### 4.6. Horizontal Joint Behavior

**Figure 16:**
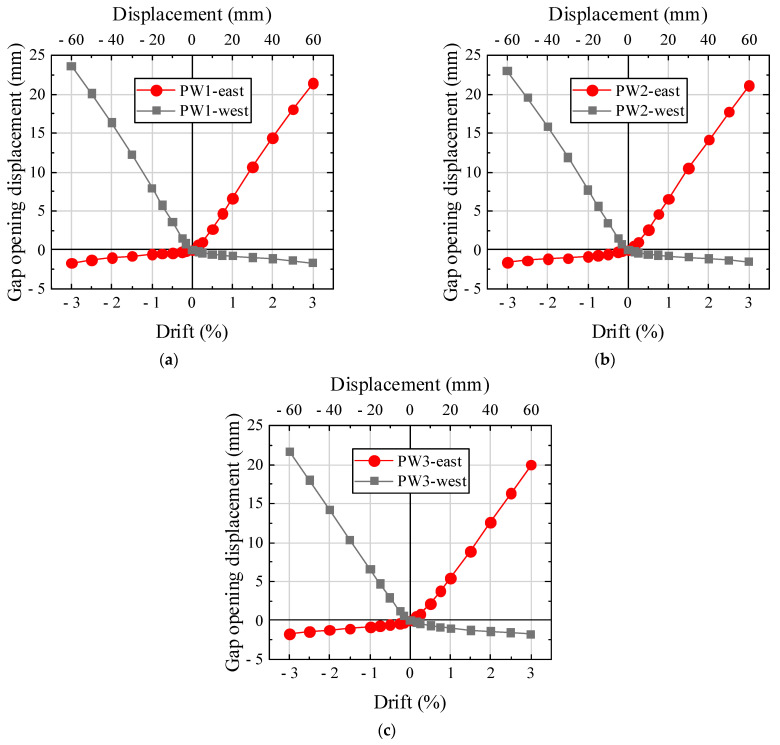
Vertical displacements. (**a**) PW1. (**b**) PW2. (**c**) PW3.

Figure 16 depicts the vertical gap opening dimensions in the horizontal panel–foundation joints at the westernmost and easternmost sites of the precast walls. The vertical displacements at both ends of the specimens exhibited a symmetrical behavior. The vertical gap displacement exhibited a linear correlation with the drift.

**Figure 17:**
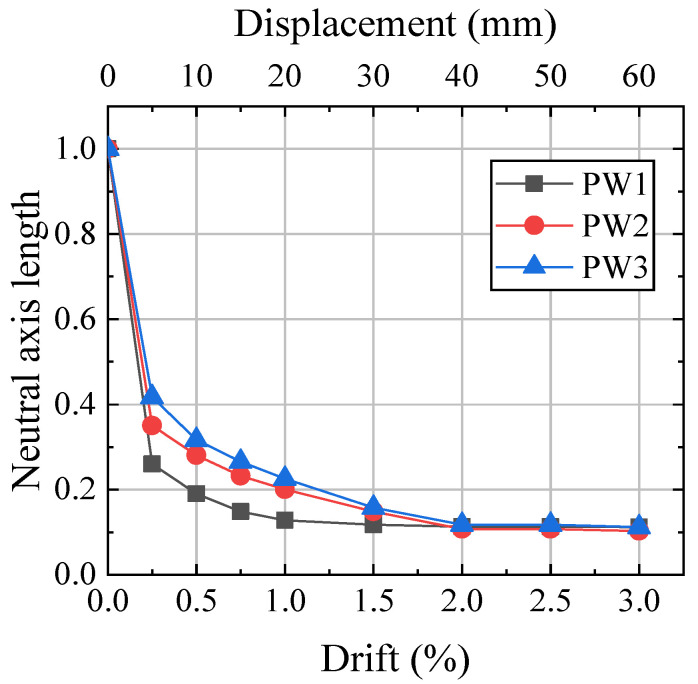
Neutral axis length.

Measured by five vertical LVDTs placed at the base of the wall, the neutral axis is the length of contact between the bottom of the wall and the foundation in the direction of the wall width. Figure 17 shows the neutral axis lengths normalized with the wall length. The neutral axis length closely matched the wall width during the initial stage of wall loading and decreased rapidly once the gap began to open. Then, the neutral axis length decreased slowly and remained almost steady after 2% drift. Owing to no concrete crushing of the wall toes, the neutral axis length did not increase at large drift. At large drift, the neutral axis length of the wall without steel jackets increased due to concrete crushing at the wall toes [9,10].

### 4.7. PT Tendon Behavior

**Figure 18:**
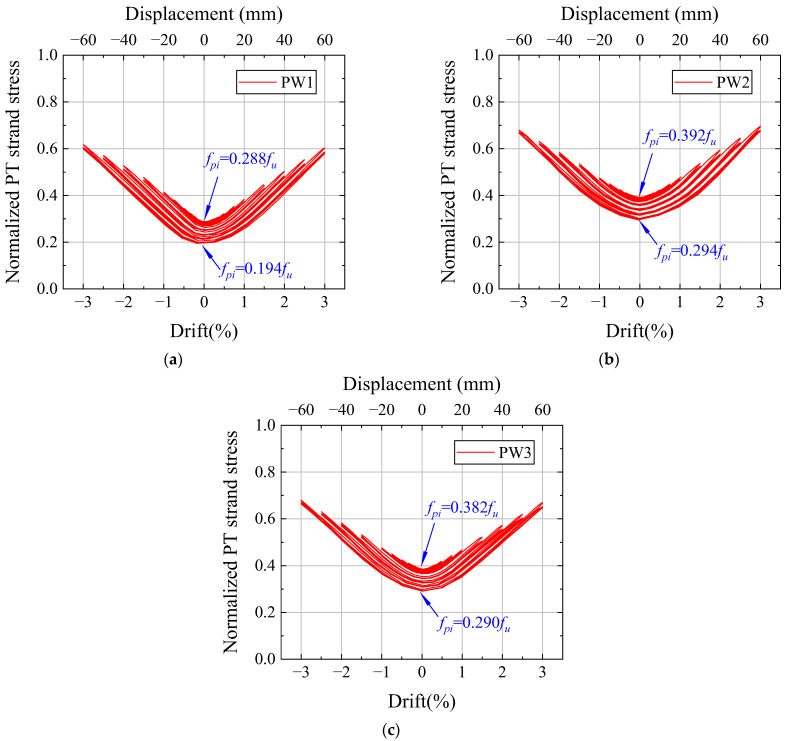
PT tendon stresses. (**a**) PW1. (**b**) PW2. (**c**) PW3.

Figure 18 illustrates the relationship between the PT tendon stress and the drift, where the PT tendon stress is taken as the standardized average stress. Owing to the arrangement of PT tendons in the center of the wall, the PT tendon stress was symmetrically distributed. Furthermore, the PT tendon achieved a maximum value of 0.73*f_u_*, confirming its state within the elastic range. The loss of PT tendon stress increased with increasing drift because the PT force increased with increasing drift, resulting in large plastic compression deformation of the anchor.

## 5. Analysis Method

To further promote the application of self-centering precast composite walls with SPFDs, the calculation formula for the horizontal lateral force of the wall was derived.

**Figure 19:**
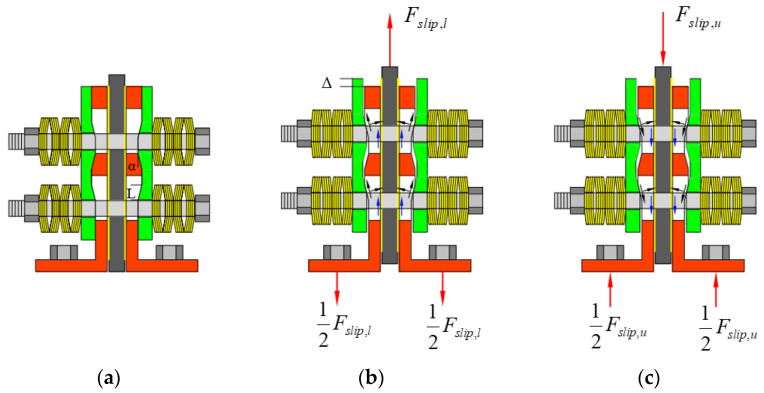
Sliding mechanism of the SPFDs. (**a**) Initial state. (**b**) Sliding at the sloped part during loading stage. (**c**) Sliding at the sloped part during unloading stage.

When the horizontal joint began to open under an earthquake, sliding would develop between the EFP and the LFP, as well as between the LFP and the web, as shown in Figure 19.

As shown in Figure 19b, as the opening of the gap at the wall–foundation connection, the EFP will slide along the sloped plate of the LFP and compress the CBS, causing it to generate greater pressure. The corresponding friction force, *F_slip,l_*, can be determined by the static equilibrium as follows:

(1) $$F_{slip,l}=2n_{b}F_{N}\times \left(\mu_{L-w}+\frac{\tan\alpha +\mu_{L-E}}{1-\mu_{M-w}\tan\alpha }\right)$$

where *n_b_* represents the number of bolts (in Figure 19, *n_b_* = 4). *F_P_*_0_ indicates the prestressing clamping load applied to the bolts, *μ_L-w_* indicates the friction coefficients for the LFP–web interfaces, and *μ_L-E_* indicates the friction coefficients at the LFP-EFP interfaces. The clamping load in the bolts, *F_N_*, is calculated as follows:

(2) $$F_{N}=F_{\Delta }+F_{p0}$$

where *F*_Δ_ represents the added clamping load of the bolts. *F*_Δ_ is given by Equation (3), where *K*_CBS_ represents the stiffness of the CBS on the bolt. Δ represents the sliding distance of the EFP on the LFP, and *α* represents the ridge angle.

(3) $$F_{\Delta }=K_{\mathrm{CBS}}\Delta \tan\alpha$$

As shown in Figure 19c, the friction force under unloading on the sloped part, *F_slip,μ_*, can be determined by the static equilibrium as follows:

(4) $$F_{slip,u}=2n_{b}F_{N}\times \left(\mu_{L-w}+\frac{\tan\alpha -\mu_{L-w}}{1+\mu_{L-w}\tan\alpha }\right)$$

To ensure that the friction device can be reused, the LFPs should maintain elasticity. Therefore, the minimum cross-sectional area of the LFPs, *A_L_*, should meet Equation (5).

(5) $$A_{L}\geq F_{slip,max}/\sigma_{y}$$

where *F_slip,max_* represents the maximum friction force under loading of the LFPs, which can be calculated using Equation (4), and *σ_y_* is the yield strength of steel.

To ensure that the disc spring maintains elasticity, *L* should meet Equation (6) as follows:

(6) $$L\leq h_{75\%}/\sin\alpha$$

where *h*_75%_ represents 75% of the maximum compression of the CBS.

As the horizontal force of the wall gradually increases, the compressive stress on one side of the wall gradually decreases, and the static friction force of the SPFD is generated and gradually increases. When the maximum static friction is reached, that is, the lateral force of the precast wall reaches the opening force, the horizontal joint starts to open, and the length of the neutral axis decreases rapidly. The opening force, *F_O_*, is calculated as follows:

(7) $$F_{O}=F_{V}(l_{\mathrm{PT}}-c/3)/H_{w}+F_{slip}\left(l_{\mathrm{SPFD}}-c/3\right)/H_{w}$$

where *F_V_* indicates the total vertical load applied by PT tendons *F*_PT_ and self-weight *N_w_*; *F_slip_* represents the friction force of the SPFD; *l*_PT_ represents the distance from the PT tendons to the edge of the steel wall toe; *l*_SPFD_ represents the length from the SPFD to the outer edge of the compressed steel wall toes; *H_w_* indicates the wall height; *c* represents the length of the neutral axis (according to the test, the neutral axis length can be taken as 0.35 *l_w_* when opening); and *l_w_* indicates the wall width.

Due to the low height of the test specimen, the length of the force arm at the resultant force point of PT tendons and gravity was similar when the wall was rocking. Therefore, it is assumed that both force arms were the same in this paper. For a multi-story building, at small drift, the plumb line of the wall gravity was on the inner side of the wall. As the drift increased, the distance between the gravity and the resultant force point gradually decreased. Gravity and PT force together provided the wall with self-centering ability. During large drift, the plumb line of the wall gravity would be on the outside of the wall, and the distance between the gravity and the resultant force point increased with the increase in the drift. Gravity hindered the self-centering effect of the PT force. Due to gravity being much weaker than PT force, the effect of gravity on self-centering was much smaller than that of PT force.

**Figure 20:**
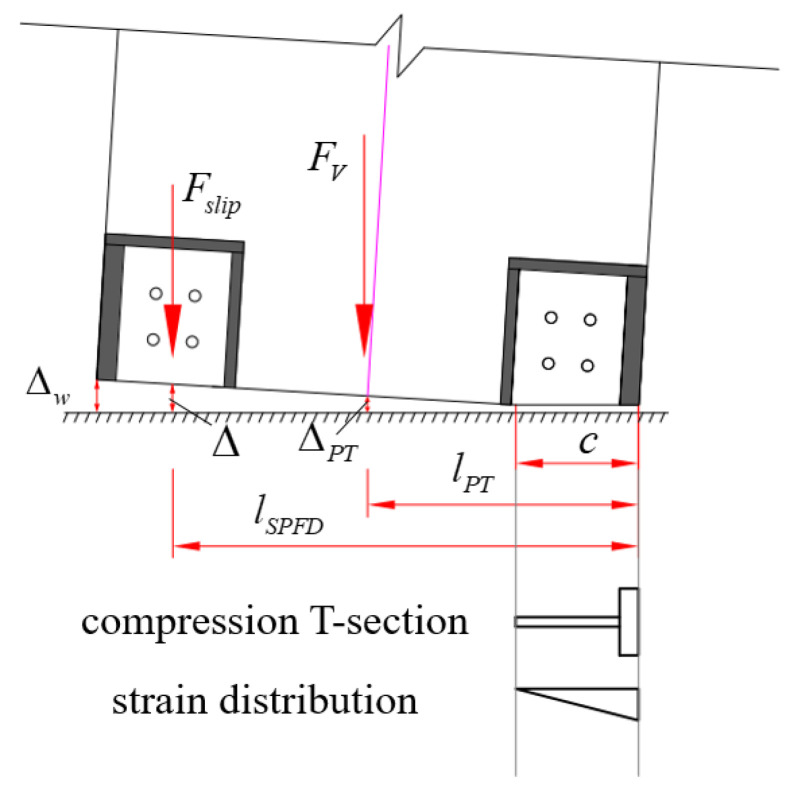
Compression T-section and strain distribution.

When the wall reaches the maximum drift, it reaches its maximum lateral force, the length of the neutral axis tends to be stable (0.08 *l_w_*), and the pressure on the wall is completely borne by the steel wall toes (Figure 20). The maximum force, *F_max_*, is calculated as follows:

(8) $$F_{\max}=\left[F_{V}\left(l_{\mathrm{PT}}-d\right)+F_{slip}\left(l_{\mathrm{SPFD}}-d\right)\right]/H_{w}$$

where *d* indicates the length from the outer edge of the wall toes under compression to the resultant point.

**Figure 21:**
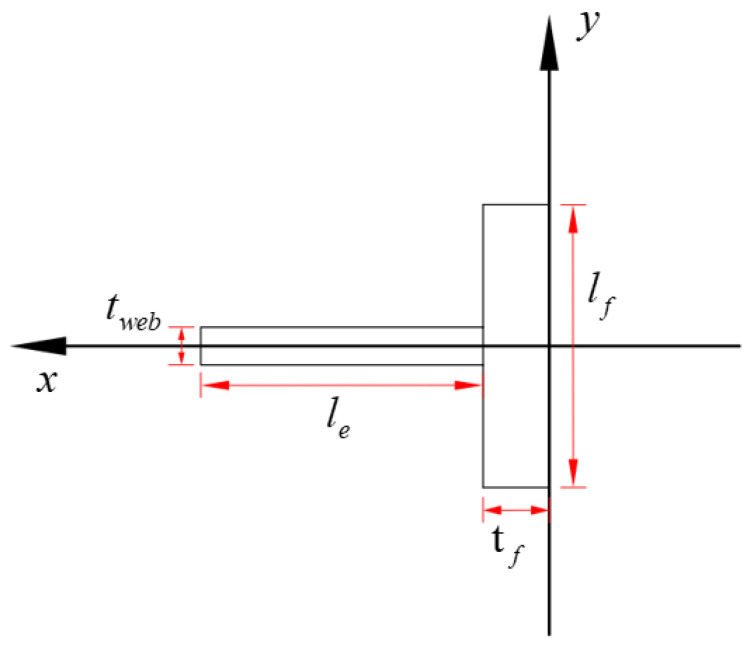
Compression section.

The distance *d* can be computed by

(9) $$d=\frac{2{\left(l_{e}+t_{f}\right)}^{3}\left(l_{e}+l_{f}\right)t_{web}+2\left(2l_{e}+t_{f}\right)\left(l_{f}-t_{web}\right)\left(2l_{e}+l_{f}\right)t_{f}^{2}}{6{\left(l_{e}+t_{f}\right)}^{2}\left(l_{e}+l_{f}\right)t_{web}+6\left(l_{e}+l_{f}\right)\left(2l_{e}+t_{f}\right)\left(l_{f}-t_{web}\right)t_{f}}$$

where *t_web_* and *t_f_* are the thickness of the web and flange (Figure 21), respectively; *l_e_* and *l_f_* represent the length of the web and flange, respectively.

The PT force, *F*_PT_, is expressed as follows:

(10) $$F_{\mathrm{PT}}=F_{\mathrm{PT},0}+K\Delta l$$

where *F*_PT,0_ and *K* denote the initial force and stiffness of the PT tendons, respectively; Δ*l* denotes the tensile length of the PT tendons, which is shown to be equal to

(11) $$\Delta l=\frac{\frac{l_{w}}{2}-c}{l_{w}-c}\Delta_{w}-a$$

where *a* denotes the anchorage deformation and shrinkage of the PT tendons; Δ*_w_* is the vertical displacement of the outer edge of the wall toes, which is shown to be equal to

(12) $$\Delta_{w}=\frac{l_{H}(l_{w}-c)}{H}$$

where *l_H_* denotes the horizontal displacement at the loading point.

Based on the relationship between PT tendon stress and the lifting height of the middle part of the wall bottom, the formula for the anchor deformation and PT tendon shrinkage, *a*, can be expressed as *a* = 0.075 *l_H_*.

The friction–slip distance, Δ, is shown to be equal to the following:

(13) $$\Delta =\frac{l_{H}(l_{\mathrm{SPFD}}-c)}{H_{w}}$$

The above formula for the wall’s lateral force was applicable to walls where the PT tendons were not broken and the concrete was not crushed. To prevent the breakage of PT tendons, the tensile length of the PT tendons Δ*l* should meet the following conditions:

(14) $$\Delta l<\varepsilon_{\mathrm{PT}y}H_{\mathrm{PT}}$$

where *ε*_PT*y*_ represents the yield strain of the PT tendons and *H*_PT_ represents the total unbonded length of the PT tendons.

**Figure 22:**
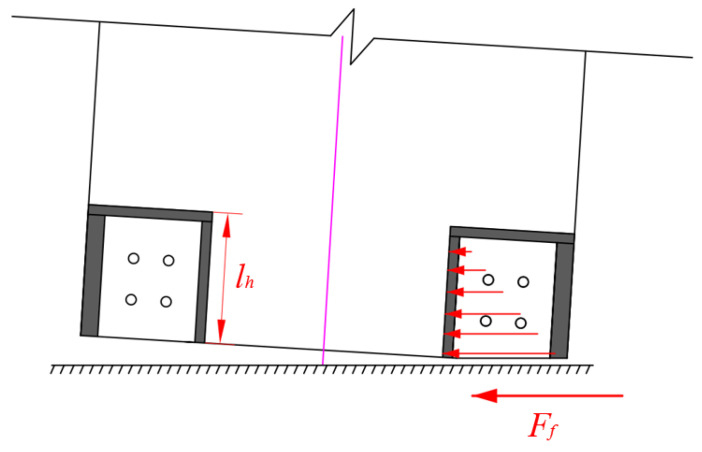
Stress distribution transmitted from steel wall toe to concrete.

As the wall was rocking, the foundation transferred the friction force *F_f_* to the steel wall toe, and then the steel wall toe transferred the friction force to the concrete. The stress distribution transmitted from the steel wall toe to the concrete is shown in Figure 22. To prevent concrete at the bottom of the wall crushing before the PT tendons yield, the stress of the concrete at the bottom of the wall should meet the following requirements:

(15) $$\frac{2F_{f,y}}{l_{f}l_{h}}<f_{c}$$

where *F_f,y_* represents the friction force when the PT tendons yield, *l_h_* represents the height of the lower part of the steel wall toes, and *f_c_* indicates the compressive strength of concrete.

The friction force, *F_f,y_*_,_ is calculated as follows:

(16) $$F_{f,y}=\left(F_{\mathrm{PT}y}+N_{w}+F_{slip,y}\right)\mu$$

where *F*_PT*y*_ indicates the force of PT tendons at yield, *F_slip,y_* represents the friction force provided by the SPFDs at the yield of the PT tendons, and *μ* indicates the friction coefficient between steel and steel. To prevent accidental brittle collapse of the wall due to crushing caused by augmented vertical stress, the axial compression ratio of the wall at the maximum drift should not exceed 0.6 [45].

By analyzing the hysteresis curves, it can be found that when the drift of the wall is 0.25% and 3%, the opening force, *F_O,the_*, and the maximum force, *F_max,the_*, are reached, respectively. The corresponding theoretical lateral forces calculated according to Equations (7) and (8) are shown in Table 3. Accordingly, the experimental lateral forces *F_O,exp_* and *F_max,exp_* are also listed in Table 3. To compare the error between the theoretical and experimental values, the absolute value of the difference is divided by the theoretical value *η* ($\eta =\left|\frac{F_{exp}-F_{the}}{F_{the}}\right|$).

The theoretical value is close to the test value, indicating that the theoretical results can provide a reference for engineering applications.

## 6. Summary and Conclusions

This paper introduces a novel self-centering precast composite wall with SPFDs. A cyclic loading test of three precast walls was conducted, and lateral force was determined by theoretical approaches. The following conclusions can be drawn:

1. The maximum residual drift of the self-centering walls with SPFDs was 0.28%, demonstrating great self-centering capacity. The energy dissipation capacity of the wall met the requirements with a relative energy dissipation ratio greater than 0.125, indicating great energy dissipation capacity. The damage to the walls was slight, meaning they exhibited great low-damage characteristics.
2. Due to the strengthening effect of the steel wall toes, the bearing capacity of the wall remained increased, and the neutral axis length did not increase significantly even at large drift, distinguishing it from walls without steel jackets. Moreover, the increasing friction force of the SPFDs with drift contributed to improving energy dissipation capacity compared to both self-centering walls without dampers and walls with flat plate friction dampers.
3. The SPFDs remained undamaged at large drift, unlike metal dampers which were prone to damage under similar conditions. Consequently, the bearing capacity, energy dissipation capacity, and residual drift of the wall with SPFDs did not decrease at large drift.
4. The initial PT force obviously influenced the residual drift and horizontal bearing capacity. Owing to a 57% higher initial PT force in PW2 compared to PW1, PW2 demonstrated a 13% reduction in residual drift and a 16% increase in bearing capacity relative to PW1.
5. The initial prestressing clamping force significantly influenced the energy dissipation capacity. The initial prestressing clamping force of PW3 was twice that of PW2, resulting in the relative energy dissipation ratio of PW3 being 1.2 times that of PW2.
6. The theoretical analysis of the friction of the SPFDs and lateral force of the self-centering wall was carried out and compared with the experimental results. The method can predict the horizontal bearing capacity of the walls reasonably well.
7. Future research should be performed to evaluate the effect of the combined form of CBS and the dimensions of steel wall toes on the seismic behavior of the walls. Furthermore, the seismic behavior and structural design method of self-centering wall structure should be studied.

## Figures and Tables

**Table 1:** Specimen descriptions.

Specimen	*F_PT_*_,0_ (kN)	*T* (N·mm)	*μ_N_*
PW1	300	44	0.102
PW2	407	44	0.137
PW3	398	88	0.134

Note: *F_PT_*_,0_ is the initial post-tensioning (PT) force; *T* is the initial torque applied to the friction bolts; *μ_N_* is the axial compression ratio (total axial force from self-weight plus the PT force divided by the wall’s effective cross-sectional area and the concrete compressive strength).

**Table 2:** Mechanical behaviors of the steel plates, PT tendons, bolts, and reinforcing bars.

Material	Type	Yield Strength (MPa)	Tensile Strength (MPa)
Steel plates	Q345	400	530
PT tendons	1860 grad	1670	1860
Bolts	10.9 grad	900	1000
Reinforcing bars	HRB 400	430	590

**Table 3:** Test and theoretical results.

Specimen		PW1	PW2	PW3
*F_O_*	*F_O,exp_* (kN)	77.24	92.07	93.92
	*F_O,the_* (kN)	82.14	96.45	101.14
	*η_O_*	6.34%	4.76%	7.69%
*F_max_*	*F_max,exp_* (kN)	231.76	268.76	295.77
	*F_max,the_* (kN)	233.28	255.21	280.40
	*η_max_*	0.66%	5.04%	5.19%

## Data Availability

Data are contained within the article.
